# Economic Justification Analysis of Minimally Invasive versus Conventional Aortic Valve Replacement

**DOI:** 10.3390/ijerph20032553

**Published:** 2023-01-31

**Authors:** Marko Jovanovic, Igor Zivkovic, Milos Jovanovic, Ilija Bilbija, Masa Petrovic, Jovan Markovic, Ivana Radovic, Ana Dimitrijevic, Ivan Soldatovic

**Affiliations:** 1 Institute for Cardiovascular Diseases “Dedinje”, 11000 Belgrade, Serbia; 2 Faculty of Medicine, University of Belgrade, 11000 Belgrade, Serbia; 3 Cardiac Surgery, University Clinical Center of Serbia, 11000 Belgrade, Serbia; 4 Faculty of Dental Medicine, University of Belgrade, 11000 Belgrade, Serbia; 5 Transfusiology Clinic, University Clinical Center of Serbia, 11000 Belgrade, Serbia; 6 Institute of Medical Statistics and Informatics, Faculty of Medicine, University of Belgrade, 11000 Belgrade, Serbia

**Keywords:** healthcare economics and organizations, heart valve prosthesis implantation, cardiac surgical procedures, aortic valve stenosis

## Abstract

There is no definitive consensus about the cost-effectiveness of minimally invasive aortic valve replacement (AVR) (MI-AVR) compared to conventional AVR (C-AVR). The aim of this study was to compare the rate of postoperative complications and total hospital costs of MI-AVR versus C-AVR overall and by the type of aortic prosthesis (biological or mechanical). Our single-center retrospective study included 324 patients over 18 years old who underwent elective isolated primary AVR with standard stented AV prosthesis at the Institute for Cardiovascular Diseases “Dedinje” between January 2019 and December 2019. Reintervention, emergencies, combined surgical interventions, and patients with sutureless valves were excluded. In both MI-AVR and C-AVR, mechanical valve implantation contributed to overall reduction of hospital costs with equal efficacy. The cost-effectiveness ratio indicated that C-AVR is cheaper and yielded a better clinical outcome with mechanical valve implantation (67.17 vs. 69.5). In biological valve implantation, MI-AVR was superior. MI-AVR patients had statistically significantly higher LVEF and a lower Euro SCORE than C-AVR patients (Mann–Whitney U-test, *p* = 0.002 and *p* = 0.002, respectively). There is a slight advantage to MI-AVR vs. C-AVR, since it costs EUR 9.44 more to address complications that may arise. Complications (mortality, early reoperation, cerebrovascular insult, pacemaker implantation, atrial fibrillation, AV block, systemic inflammatory response syndrome, wound infection) were less frequent in the MI-AVR, making MI-AVR more economically justified than C-AVR (18% vs. 22.1%).

## 1. Introduction

Aortic valve stenosis is the most prevalent entity of valvular heart diseases, especially among patients older than 65 years of age, affecting 2–7% of the advanced age population [1]. Conventional surgery of the aortic valve through median sternotomy has long been regarded as the gold standard due to excellent long-term results and a low mortality rate (1–3%) [1,2]. As a consequence of the rise in life expectancy, older patients more frequently require aortic valve replacement (AVR) due to the fact that prevalence increases with age [1]. These patients often have higher body mass index and comorbidities, e.g., diabetes, hypertension, chronic obstructive pulmonary disease, cerebrovascular disease, and renal failure, which render them high-risk candidates for conventional AVR (C-AVR) [3]. The attempt to elude risks of median sternotomy has led to the development of new surgical techniques, transcatheter aortic valve implantation (TAVI), and less or minimally invasive surgical approaches. TAVI enables surgeons to avoid risks of sternotomy, extracorporeal circulation, and cross-clamping of the aorta but is associated with increased pacemaker implantation rates, pericardial tamponade, stroke, pulmonary embolism, and perivalvular leak [4,5]. The partial J-shaped upper ministernotomy and right thoracotomy (minimally invasive approach), besides being less traumatic for the patient, are also associated with a shorter hospital stay, faster recovery, and lower rates of transfusion and wound infection [6,7]. Downfalls of these techniques are longer cardiopulmonary bypass (CPB) and aortic cross-clamp times [8,9]. At present, there is no definitive consensus about the cost-effectiveness of minimally invasive AVR (MI-AVR) compared to the conventional approach. Some authors report lower total hospital costs for minimally invasive surgery, but others reported opposite findings [8,10]. In a multi-center study including 17 U.S.-based hospitals, it was concluded that patients undergoing MI-AVR compared to C-AVR were three times more frequently discharged by the fourth postoperative day, and fewer MI-AVR patients received a blood product transfusion (24.6% vs. 31.8%) [10]. These two factors were considered to be key influencing factors that were attributed to lower hospital costs for MI-AVR patients by 5% [10]. Conversely, a single-centered study conducted in a United-Kingdom-based hospital showed that costs during the index admission were significantly greater in the MI-AVR group, which were primarily influenced by increased time in the operating room (a GBP 1140 mean difference) [8].

These differences regarding hospital expenses may reflect the differences between the different healthcare systems where the analyses were performed. Unlike similar studies that have been previously performed, this study is the first to our knowledge to perform such an analysis comparing total hospital costs and outcomes between MI-AVR and C-AVR in a middle-income country featuring a comprehensive universal healthcare system. Furthermore, in Serbia, cardiovascular diseases (CVD) are the leading cause of death (51.8% of all deaths), with a trending increase in mortality over the past decade. CVD poses a significant economic burden on the healthcare system and overall is a public health problem that needs to be focused on through education and prevention programs.

The aim of this study is to compare the rate of postoperative complications and the total hospital cost of MI-AVR compared to C-AVR in general and with regard to the type of aortic prosthesis used.

## 2. Materials and Methods

### 2.1. Study Design

The study was designed as a single-center observational retrospective study conducted at the Institute for Cardiovascular Diseases “Dedinje”. Our study included 324 patients who underwent elective isolated primary AVR between January 2019 and December 2019.

The study was approved by the Ethical Committee of the Institute for Cardiovascular Diseases “Dedinje” (no. 655), and the local ethics committee waived the need for patient written consent to be included in our study due to the retrospective nature of the investigation.

Our study included all patients over 18 years of age undergoing elective open surgical aortic valve replacement (SAVR), with standard stented aortic valve prosthesis, both mechanical and biological. Exclusion criteria were reintervention (reoperation), emergency procedures, combined surgical interventions, and patients with sutureless valves (Perceval S, LivaNova PLC, London, UK). The decision on the type of surgical approach was made by the surgeon on the basis of their experience and preference, as well as the patient’s preferences.

Patient records were classified according to the operative approach: full-length median sternotomy—conventional AVR (C-AVR, group 1) versus either partial J-shaped upper ministernotomy or right anterior thoracotomy (RAT) (minimally invasive, MI-AVR, group 2). In group 1, there were 235 patients, while group 2 consisted of 89 patients. Of the 89 patients in the MI-AVR group, only 21 patients had a RAT without the use of a knotting system and therefore were combined in the group with the patients undergoing partial J-shaped upper ministernotomy for the purpose of our analysis. Both groups were further divided according to the type of implanted AV (biological or mechanical aortic prosthesis).

Data on patient preoperative characteristics and clinical outcomes were retrieved from the patients’ hospital records. Data on total hospital cost was obtained from the Financial service of the Institute for Cardiovascular Disease “Dedinje” defined by the Republic’s Fund for Health Insurance. Due to the retrospective nature of our study, patients and the public were not involved in the design of our study.

### 2.2. Surgical Techniques

Arterial cannulation of ascending aorta and venous cannulation of the right atrial appendage were made centrally through the surgical site in almost all cases (99.8% of patients). The left ventricle was vented through the right superior pulmonary vein. Cold blood cardioplegia was used for myocardial protection, and the pericardial cavity was flooded with CO_2_. Conventional median sternotomy was performed through a midline incision from the sternal notch 20–25 cm long, followed by the full-length sternotomy. In partial J-shaped upper ministernotomy, an incision was made from the sternal angle to the fourth intercostal space. The sternum was opened from the sternal angle to the third or fourth intercostal space. Right thoracotomy was performed through the second or third right intercostal space. Standard biological or mechanical aortic prostheses were implanted in the usual manner [11,12,13].

### 2.3. Outcomes Recorded

In this study, the outcomes of surgical procedures were monitored only during in-hospital treatment. Outcomes were recorded with respect to the rate of post-operative complications (mortality, early reoperation, cerebrovascular insult, pacemaker implantation, atrial fibrillation, AV block, systemic inflammatory response syndrome (SIRS), wound infection) and hospital cost.

For pharmacoeconomic analysis, the total hospital costs for each operated patient served as the initial parameter. The effectiveness of the procedure was assessed by the percent of patients who had no complications. Incremental effectiveness was calculated as the difference in the effectiveness between the given groups. The results of the pharmacoeconomic analysis were presented as the cost-effectiveness ratio (CER) and incremental cost-effectiveness ratio (ICER). CER is calculated as the ratio of the cost of each individual therapy (procedure) and its therapeutic effect. ICER is calculated as the ratio of the cost difference of two alternative therapies and the difference in effectiveness between the compared therapies. ICER, in fact, shows the additional costs per unit of additional effectiveness. Outcomes were analyzed separately according to the surgical technique as well as by the type of valve used.

$$\mathrm{CER}=\frac{\mathrm{Cos}t\;\mathrm{of}\;\mathrm{each}\;\mathrm{individual}\;\mathrm{procedure}\;\left(\mathrm{EUR}\right)}{\mathrm{Therapeutic}\;\mathrm{Effect}\;}$$

$$\mathrm{ICER}=\frac{\mathrm{Cos}t\;\mathrm{difference}\;\left(\mathrm{EUR}\right)}{\mathrm{Difference}\;\mathrm{in}\;\mathrm{Outcome}\;}$$

### 2.4. Statistical Analysis

Results are presented as count (%) or mean ± standard deviation, depending on the data type and distribution. Normal distribution was tested using the Kolmogorov–Smirnov test, as well as visual analysis of histogram of frequencies and Q-Q plot. Groups were compared using parametric (*t*-test) and nonparametric (chi-squared, Mann–Whitney U-test) tests. All *p*-values less than 0.05 were considered significant. All data were analyzed using SPSS 29.0 (IBM Corp. Released 2022. IBM SPSS Statistics for Windows, Version 29.0. Armonk, NY, USA: IBM Corp.).

## 3. Results

Our study included a total of 324 patients, 235 patients (72.5%) with C-AVR, and 89 patients (27.5%) with MI-AVR (Table 1).

The average age of all patients was 65 ± 11 (22–87) years. The group with C-AVR was dominated by men, while the distribution of men and women was significantly more uniform in the group with MI-AVR.

The prevalence of comorbidities, which could compromise the assessment of clinical outcomes and hospital treatment costs, did not significantly differ between groups.

The most common comorbidity recorded was hypertension, followed by coronary artery disease and hyperlipidemia (Table 1).

Compared to the C-AVR group, patients from the MI-AVR group had a statistically significantly higher left ventricular ejection fraction (LVEF) and a statistically significantly lower Euro SCORE II (Table 1).

Fatal outcome was registered in two patients, one in each group.

The occurrence of CVI was registered only in the C-AVR group. The need for pacemaker implantation and the occurrence of SIRS was very rare and was uniformly represented in both groups of patients (Table 1).

In the C-AVR group, a mechanical prosthesis was used almost three times more often when compared to the biological prosthesis (173:62); in the group of patients undergoing MI-AVR, this number was almost equal (45:44).

In patients with mechanical prosthesis, there was a statistically significant difference in the average age between the C-AVR and MI-AVR groups (*p* < 0.001).

Compared to the C-AVR group, patients with mechanical prosthesis from the MI-AVR group had a statistically significantly higher LVEF and lower Euro SCORE II. Furthermore, the occurrence of CVI, SIRS, and pacemaker implantation was registered only in the C-AVR group. However, fatal outcome was registered only in the minimally invasive group (Table 2).

The analysis of demographics of patients with biological prostheses showed a statistically higher percentage of males in the conventional group. In the minimally invasive group, both genders were equally represented. Opposite to patients with mechanical valves, there was no significant difference in LVEF and Euro SCORE II between patients with biological prostheses.

Fatal outcome and CVI only occurred in C-AVR patients with biological prostheses (Table 3).

The total hospital costs per patient, the efficiency of surgical procedures, and the method of calculating ICER for the two main groups of patients (all patients) and their subgroups (mechanical prosthesis and biological prosthesis) are shown in Table 4.

If the two surgical procedures (C-AVR and MI-AVR) are considered as a whole (all patients), it can be concluded that the total hospital costs, but also their usefulness, were approximately equal. A slight advantage was found for the MI-AVR procedure, since in the case of the C-AVR surgical procedure, EUR 9.44 more must be given to address complications that may arise, compared to the MI-AVR procedure.

The sections of Table 4 denoted as mechanical prosthesis and biological prosthesis refer to the cost-effectiveness analysis after the implantation of mechanical or biological prosthesis separately.

When comparing the two surgical approaches by prosthesis type in terms of total hospital costs and effectiveness, the MI-AVR approach was consistently less costly and more effective when compared across all patients and when a biological prosthesis was used; however, when mechanical prosthesis was used, the effectiveness was only slightly higher in C-AVR versus MI-AVR, but not significantly (81.5% vs. 80%).

In the case of mechanical valve implantation, the CER showed that C-AVR is cheaper and yields better clinical outcomes during hospital follow-up, but not significantly (81.5% vs. 80%). Furthermore, mechanical valve implantation contributed to the reduction of hospital costs by EUR 85.33 per patient without significantly compromising efficacy (Table 4).

Table 5 shows the hospital cost data caused by the occurrence of some important clinical complications following aortic valve replacement. The analysis was performed for the whole group of patients (N = 324) due to the relatively rare occurrence of serious clinical complications.

The implantation of a pacemaker increased costs by EUR 2399.73, and slightly higher costs (EUR 3879.33) can be expected in the case of surgical revision. An episode of SIRS led to an even higher increase in costs (EUR 4069.99), but the maximum increase in costs was recorded after the stroke (EUR 12,818.49 per episode). The data refer to patients who had only one of the complications. In the case of associated complications, these costs will certainly be much higher.

There was a statistically significant difference in the material cost, in the total, and if the mechanical prosthesis had been implanted. Table 6 shows that material cost was significantly higher in patients treated with MI-AVR. The Republic’s Fund for Health Insurance costs for all patients were higher in patients treated with MI-AVR, but there was no statistical significance.

## 4. Discussion

Aortic valve replacement has been widely accepted as the most effective treatment for aortic stenosis and aortic regurgitation [14]. Over the years, newer surgical approaches have been developed to improve safety and efficacy.

Many have criticized the minimally invasive approach for AVR as being more complex, technically demanding, and costly due to the increased operative time and equipment needed and that it may be associated with an increased risk for complications due to the perceived longer cross-clamp and bypass times; however, these studies have not shown that cross-clamp and bypass time have been associated with an increased risk of morbidity and mortality to the level of statistical significance [7,15,16,17]. Furthermore, MI-AVR has been associated with decreased blood product utilization, which has been associated with better short- and long-term outcomes following cardiac surgery [18,19,20]. As a result, MI-AVR resulted in more favorable short- and long-term outcomes, decreased hospital stay, lower blood product utilization, and therefore ultimately decreased cost. Similarly, in our study, when we compared total hospital costs and outcomes of C-AVR and MI-AVR, we found that there was a slight advantage in the MI-AVR approach with regard to overall efficacy as well as a decreased total hospital cost by EUR 9.44 (Table 4). With regard to surgical complications, patients who underwent C-AVR were nearly twice as likely to have complications than MI-AVR when biological prosthesis was used (32.3% vs. 15.9%, *p* = 0.057). Each type of complication can carry a significant cost for not only the hospital but for the patient as well, and more importantly can impact the patient’s post-operative recovery.

Some studies have reported that MI-AVR reduced the total recovery period by nearly a month when compared to the conventional approach, and other similar single-center studies performed by Cohn et al. and Cosgrove et al. found that MI-AVR reduced hospital costs directly by 19% or an average of USD 7000 [7,21,22]. Another study by Ghanta et al. found that the minimally invasive approach decreased costs by 5% and also noted, similar to our study, that mortality and morbidity outcomes of MI-AVR and C-AVR were similar [10]. Furthermore, similar to our study, the study conducted by Ghanta et al. also yielded decreased total hospital costs for MI-AVR [23]. Namely, in our study, there was a 14.97% reduction in total hospital costs for MI-AVR compared to C-AVR; however, this was only if biological prosthesis was used. In the case of mechanical prosthesis, there was a slight advantage in efficacy and cost in the C-AVR group, despite the fact that the patients in the MI-AVR group were younger (56.8 ± 12.5 versus 63.9 ± 11.1) compared to the patients in the C-AVR group (*p* < 0.001). This difference, however, should be further explored in future studies since mechanical prosthesis was used almost four times more often in the C-AVR patients compared to MI-AVR and three times more often than biological prosthesis in the C-AVR group.

Overall, the MI-AVR approach is more favorable in terms of the decreased cost, shorter hospital stay, and significantly shorter recovery period [7,23].

One of the limitations of this study might be its observational nature, as well as how the decision was made for the type of surgical approach, making it susceptible to patient selection bias. Moreover, this is a single-center, retrospective study that does not provide a complete insight into the real-world application of the differences between the two approaches. Furthermore, our calculations only take into consideration direct hospital costs disregarding hospital costs for rehabilitation and leave of absence financial losses. Eventually, the decreased hospital stay would lead to less loss of productivity; however, future studies should look further into out-of-hospital expenses across multiple centers in different countries to determine the efficacy and cost-effectiveness in a real-world setting. In addition, another limitation to our study is the fact that in our study population C-AVR was performed three times more often than MI-AVR, and that RAT without the use of a knotting system was combined with the parital J-shaped ministerotomy approach to make up the MI-AVR group. Given the challenges associated with the RAT approach, studies should split each surgical approach separately with an adequate number of patients to achieve a powerful analysis and consider exploring how the use of a knotting system in RAT may change the overall pharmacoeconomic analysis. Future studies could consider comparing outcomes on the basis of the surgeon’s experience with each respective approach, matching patients in each group on the basis of age, risk factors, and level of aortic stenosis and then comparing overall costs (hospital and out of hospital costs) and outcomes and analyzing how the use of a knotting system in RAT may affect results.

The difference in costs for implantation of mechanical versus biological valves (mechanical valves cheaper in the C-AVR group and biological valves cheaper in the MI-AVR) is difficult to explain, particularly since the implantation of the mechanical valve resulted in a better clinical outcome and decreased costs if analyzed together. Furthermore, the MI-AVR group had less patients included (n = 89 versus n = 235), which could mean that more patients need to be included in the study to reach a more meaningful conclusion.

## 5. Conclusions

With regard to the total hospital costs per patient, our study found that MI-AVR was more cost-effective if the biological prosthesis was used, but overall, it was approximately equal in terms of costs and effectiveness compared to C-AVR. Complications were also less frequent in the MI-AVR group, making MI-AVR more economically justified when compared to the C-AVR. With respect to ICER, overall and in the biological prosthesis groups, MI-AVR was more favorable. However, when the mechanical valve was used, the ICER was slightly more favorable for the C-AVR group.

## Figures and Tables

**Table 1:** Preoperative characteristics, clinical status, and the most important clinical outcomes of conventional and minimally invasive AVR patients.

		AVR		*p*-Value
	Total (N = 324)	Conventional (N = 235)	Minimally Invasive (N = 89)	
Demographics				
Age (years)	65.7 ± 11.4	65.9 ± 10.9	64.9 ± 12.7	0.453 ^a^
Gender male	204 (63%)	155 (66%)	49 (55.1%)	0.070 ^b^
Risk factors				
Hypertension	229 (70.7%)	161 (68.5%)	68 (76.4%)	0.164 ^b^
Hyperlipidemia	40 (12.3%)	28 (11.9%)	12 (13.5%)	0.702 ^b^
CVI	27 (8.3%)	16 (6.8%)	11 (12.4%)	0.107 ^b^
CRD	21 (6.5%)	14 (6.0%)	7 (7.9%)	0.534 ^b^
PVD	25 (7.7%)	20 (8.5%)	5 (5.6%)	0.384 ^b^
Smoking	126 (38.9%)	89 (37.9%)	37 (41.6%)	0.542 ^b^
CAD	56 (17.2%)	41 (17.4%)	15 (16.9%)	0.900 ^b^
DM	42 (13%)	29 (12.3%)	13 (14.6%)	0.721 ^b^
Clinical status				
NYHA class				
I	26 (8.0%)	20 (8.5%)	6 (6.7%)	0.834 ^d^
II	237 (73.1%)	173 (73.6%)	64 (71.9%)	
III	58 (17.9%)	40 (17.0%)	18 (20.2%)	
IV	3 (8.0%)	2 (0.9%)	1 (1.1%)	
LVEF (%)	51.38 ± 10.95	50.46 ± 11.42	53.82 ± 9.20	0.013 ^c^
AVA (cm^2^)	0.67 ± 0.22	0.65 ± 0.22	0.7 ± 0.21	0.081 ^a^
MSG (mmHg)	59.2 ± 18.62	59.5 ± 19.3	58.3 ± 16.8	0.590 ^a^
Euro SCORE II	1.91 ± 2.2	2.02± 2.36	1.61 ± 1.62	0.028 ^c^
Outcomes				
Death	2 (0.6%)	1 (0.4%)	1 (1.1%)	0.475 ^d^
Revision	28 (8.6%)	17 (7.2%)	11 (12.4%)	0.143 ^b^
CVI	7 (2.2%)	7 (3%)	0	0.196 ^d^
Pacemaker implant	5 (1.5%)	3 (1.3%)	2 (2.2%)	0.618 ^d^
AF	17 (5.2%)	14 (6%)	3 (3.4%)	0.418 ^d^
AV block	6 (1.9%)	3 (1.3%)	3 (3.4%)	0.352 ^d^
SIRS	5 (1.5%)	4 (1.7%)	1 (1.1%)	1.000 ^d^
Wound infection	11 (3.4%)	9 (3.8%)	2 (2.2%)	0.734 ^d^
Complications (total)	68 (21%)	52 (22.1%)	16 (18%)	0.413 ^b^

Continuous variables are presented as mean ± standard deviation, categorical variables as N (%). CVI—cerebrovascular insult; CRD—chronic renal disease; PVD-peripheral vascular disease; CAD—coronary artery disease; DM—diabetes mellitus; NYHA—New York Heart Association; LVEF—left ventricular ejection fraction; AVA—aortic valve area; MSG—mean systolic gradient; AF—atrial fibrillation; SIRS—systemic inflammatory response syndrome. Applied tests: ^a^ Student’s *t*-test; ^b^ Pearson chi-squared test; ^c^ Mann–Whitney U test; ^d^ Fisher’s exact test.

**Table 2:** Preoperative characteristics, clinical status, and the most important clinical outcomes of conventional and minimally invasive AVR patients with mechanical prostheses.

		Mechanical Prosthesis		*p*-Value
	Total (N = 218)	Conventional (N = 173)	Minimally Invasive (N = 45)	
Demographics				
Age (years)	62.5 ± 11.7	63.9 ± 11.1	56.8 ± 12.5	<0.001 ^a^
Gender male	134 (61.5%)	109 (63%)	25 (55.6%)	0.360 ^b^
Risk factors				
Hypertension	155 (71.1%)	120 (69.4%)	35 (77.8%)	0.267 ^b^
Hyperlipidemia	27 (12.4%)	21 (12.1%)	6 (13.3%)	0.828 ^b^
CVI	17 (7.8%)	10 (5.8%)	7 (15.6%)	0.054 ^d^
CRD	15 (6.9%)	12 (6.9%)	3 (6.7%)	1.000 ^d^
PVD	15 (6.9%)	14 (8.1%)	1 (2.2%)	0.317 ^d^
Smoking	81 (37.2%)	65 (37.6%)	16 (35.6%)	0.803 ^b^
CAD	35 (16.1%)	28 (16.2%)	7 (15.6%)	0.918 ^b^
DM	29 (13.3%)	22 (12.7%)	7 (15.6%)	0.800 ^b^
Clinical status				
NYHA class				
I	19 (8.7%)	16 (9.2%)	3 (6.7%)	0.656 ^d^
II	159 (72.9%)	126 (72.8%)	33 (73.3%)	
III	38 (17.4%)	30 (17.3%)	8 (17.8%)	
IV	2 (0.9%)	1 (0.6%)	1 (2.2%)	
LVEF (%)	51.55 ± 10.80	50.30 ± 11.55	56.33 ± 4.93	0.002 ^c^
AVA (cm^2^)	0.65 ± 0.23	0.64 ± 0.23	0.69 ± 0.20	0.265 ^a^
MSG (mmHg)	59.74 ± 19.76	60.0 ± 20.3	58.8 ± 17.6	0.728 ^a^
Euro SCORE II	1.77 ± 2.15	1.93 ± 2.37	1.14 ± 0.63	0.002 ^c^
Outcomes				
Death	1 (0.5%)	0	1 (2.2%)	0.206 ^d^
Revision	15 (6.9%)	9 (5.2%)	6 (13.3%)	0.090 ^d^
CVI	4 (1.8%)	4 (2.3%)	0	0.583 ^d^
Pacemaker implant	3 (1.4%)	3 (1.7%)	0	1.000 ^d^
AF	10 (4.6%)	9 (5.2%)	1 (2.2%)	0.691 ^d^
AV block	4 (1.8%)	3 (1.7%)	1 (2.2%)	1.000 ^d^
SIRS	4 (1.8%)	4 (2.3%)	0	0.583 ^d^
Infection	9 (4.1%)	8 (4.6%)	1 (2.2%)	0.689 ^d^
Complications	41 (18.8%)	32 (18.5%)	9 (20.0%)	0.818 ^b^

Continuous variables are presented as mean ± standard deviation, categorical variables as N (%). CVI—cerebrovascular insult; CRD—chronic renal disease; PVD—peripheral vascular disease; CAD—coronary artery disease; DM—diabetes mellitus; NYHA—New York Heart Association; LVEF—left ventricular ejection fraction; AVA—aortic valve area; MSG—mean systolic gradient; AF—atrial fibrillation; SIRS—systemic inflammatory response syndrome. Applied tests: ^a^ Student’s *t*-test; ^b^ Pearson chi-squared test; ^c^ Mann–Whitney U test; ^d^ Fisher’s exact test.

**Table 3:** Preoperative characteristics, clinical status, and the most important clinical outcomes of conventional and minimally invasive AVR patients with biological prostheses.

		Biological Prosthesis		*p*-Value
	Total (N = 106)	Conventional (N = 62)	Minimally Invasive (N = 44)	
Demographics				
Age (years)	72.2 ± 7.2	71.6 ± 8.1	73.1 ± 5.7	0.307 ^a^
Gender male	70 (66%)	46 (74.2%)	24 (54.5%)	0.035 ^b^
Risk factors				
Hypertension	74 (69.8%)	41 (66.1%)	33 (75%)	0.327 ^b^
Hyperlipidemia	13 (12.3%)	7 (11.3%)	6 (13.6%)	0.717 ^b^
CVI	10 (9.4%)	6 (9.7%)	4 (9.1%)	1.000 ^d^
CRD	6 (5.7%)	2 (3.2%)	4 (9.1%)	0.230 ^d^
PVD	10 (9.4%)	6 (9.7%)	4 (9.1%)	1.000 ^d^
Smoking	45 (42.5%)	24 (38.7%)	21 (47.7%)	0.355 ^b^
CAD	21 (19.8%)	13 (21.0)	8 (18.2%)	0.723 ^b^
DM	13 (12.3%)	7 (11.3)	6 (13.6%)	0.950 ^b^
Clinical status				
NYHA class				
I	7 (6.6%)	4 (6.5%)	3 (6.8%)	0.797 ^d^
II	78 (73.6%)	47 (75.8%)	31 (70.5%)	
III	20 (18.9%)	10 (16.1%)	10 (22.7%)	
IV	1 (0.9%)	1 (1.6%)	0	
LVEF (%)	51.04 ± 11.29	50.89 ± 11.44	51.25 ± 11.62	0.682 ^c^
AVA (cm^2^)	0.69 ± 0.20	0.67 ± 0.18	0.71 ± 0.22	0.333 ^a^
MSG (mmHg)	58.09 ± 16.05	58.3 ± 16.2	57.8 ± 16.1	0.853 ^a^
Euro SCORE II	2.2 ± 2.27	2.27 ± 2.38	2.09 ± 2.13	0.255 ^c^
Outcomes				
Death	1 (0.9%)	1 (1.6%)	0	1.000 ^d^
Revision	13 (12.3%)	8 (12.9%)	5 (11.4%)	0.812 ^b^
CVI	3 (2.8%)	3 (4.8%)	0	0.265 ^d^
Pacemaker implant	2 (1.9%)	0 (0.0%)	2 (4.5%)	0.170 ^d^
AF	7 (6.6%)	5 (8.1%)	2 (4.5%)	0.697 ^d^
AV block	2 (1.9%)	0 (0.0%)	2 (4.5%)	0.170 ^d^
SIRS	1 (0.9%)	0 (0.0%)	1 (2.3%)	0.415 ^d^
Infection	2 (1.9%)	1 (1.6%)	1 (2.3%)	1.000 ^d^
Complications	27 (25.5%)	20 (32.3%)	7 (15.9%)	0.057 ^b^

Continuous variables are presented as mean ± standard deviation, categorical variables as N (%). CVI—cerebrovascular insult; CRD—chronic renal disease; PVD—peripheral vascular disease; CAD—coronary artery disease; DM—diabetes mellitus; NYHA—New York Heart Association; LVEF—left ventricular ejection fraction; AVA—aortic valve area; MSG—mean systolic gradient; AF—atrial fibrillation; SIRS—systemic inflammatory response syndrome. Applied tests: ^a^ Student’s *t*-test; ^b^ Pearson chi-squared test; ^c^ Mann–Whitney U test; ^d^ Fisher’s exact test.

**Table 4:** Total hospital cost and incremental cost-effectiveness (ICER) of operative AVR protocols.

	Total Cost (EUR)/Patient	Effectiveness (%)	CER (EUR/%)	Decremental Cost (EUR/Patient)	Incremental Effectiveness (%)	ICER (EUR)
All Patients (N = 324)						
C-AVR	6020.03	77.9	77.28			
MI-AVR	5981.31	82	72.94	−38.72	+4.1	−9.44
Mechanical Prosthesis (N = 218)						
C-AVR	5474.78	81.50	67.17			
MI-AVR	5560.11	80	69.5	+85.33	−1.5	−56.89
Biological Prosthesis (N = 106)						
C-AVR	7541.48	67.7	111.4			
MI-AVR	6412.08	84.1	76.24	−1.129.4	+16.4	−68.87

C—conventional AVR; MI—minimally invasive AVR.

**Table 5:** Hospital costs per episode of serious clinical complications.

Type of Complication	Complication Costs
Surgical revision alone	EUR 3879.33 per episode
Stroke alone	EUR 12,818.49 per episode
Pacemaker alone	EUR 2399.73 per episode
SIRS alone	EUR 4069.99 per episode

SIRS—systemic inflammatory response syndrome.

**Table 6:** Therapy costs (EUR).

	Surgical Approach		*p*-Value
All (N = 324)	Conventional (N = 235)	Minimally Invasive (N = 89)	
Service	2740.4 (2481.7–3151.2)	2672.3 (2441.9–3011.8)	0.480
Patient care	224.8 (187.3–274.9)	227.9 (193.3–278.7)	0.802
Materials	1634.7 (1450.4–2030.3)	1789.2 (1566.2–2268)	0.006
Medications	237.6 (201.3–318.7)	240 (204.7–323.9)	0.407
**Total costs**	4858.6 (4404.7–5877.8)	4991.4 (4537.4–5997.3)	0.274
**Mechanical (N = 218)**	**Conventional (N = 173)**	**Minimally Invasive (N = 45)**	
Service	2740.4 (2492.2–3083.2)	2554.7 (2409.1–2977.8)	0.180
Patient care	226.5 (187.3–266.3)	227.9 (185.3–283.3)	0.795
Materials	1587.3 (1400.8–1820.2)	1660.5 (1536–1996.8)	0.018
Medications	236.2 (202.3–306.4)	227.9 (203–282.9)	0.885
**Total costs**	4793.2 (4345.7–5541.3)	4780.2 (4502.2–5462.8)	0.564
**Biological (N = 106)**	**Conventional (N = 62)**	**Minimally Invasive (N = 44)**	
Service	2750.6 (2449.2–3712)	2744.5 (2517.5–3147.2)	0.918
Patient care	219.9 (188–355.2)	226.1 (196.3–273.9)	0.939
Materials	1939.9 (1641.2–2463.5)	1865.7 (1716.9–2532.7)	0.908
Medications	247.6 (200–375.5)	247.2 (219.2–354.3)	0.505
**Total costs**	5210.3 (4645.9–7827.1)	5170.7 (4697.6–6416.6)	0.715

Results are presented as median (25–75th percentile). Mann–Whitney U test was used for all analyses.

## Data Availability

The data presented in this study are available on request from the corresponding author. The data are not publicly available due.
